# Exploring Neuromodulation for Dynamic Learning

**DOI:** 10.3389/fnins.2020.00928

**Published:** 2020-09-18

**Authors:** Anurag Daram, Angel Yanguas-Gil, Dhireesha Kudithipudi

**Affiliations:** ^1^Neuromorphic AI Lab, University of Texas, San Antonio, TX, United States; ^2^Argonne National Labs, Lemont, IL, United States

**Keywords:** neuromodulation, ModNet, one-shot learning, dynamic learning, mushroom body output neurons (MBONs)

## Abstract

A continual learning system requires the ability to dynamically adapt and generalize to new tasks with access to only a few samples. In the central nervous system, across species, it is observed that continual and dynamic behavior in learning is an active result of a mechanism known as neuromodulation. Therefore, in this work, neuromodulatory plasticity is embedded with dynamic learning architectures as a first step toward realizing power and area efficient few shot learning systems. An inbuilt modulatory unit regulates learning based on the context and internal state of the system. This renders the system an ability to self modify its weights. In one of the proposed architectures, ModNet, a modulatory layer is introduced in a random projection framework. ModNet's learning capabilities are enhanced by integrating attention along with compartmentalized plasticity mechanisms. Moreover, to explore modulatory mechanisms in conjunction with backpropagation in deeper networks, a modulatory trace learning rule is introduced. The proposed learning rule, uses a time dependent trace to modify the synaptic connections as a function of ongoing states and activations. The trace itself is updated via simple plasticity rules thus reducing the demand on resources. The proposed ModNet and learning rules demonstrate the ability to learn from few samples, train quickly, and perform few-shot image classification in a computationally efficient manner. The simple ModNet and the compartmentalized ModNet architecture learn benchmark image classification tasks in just 2 epochs. The network with modulatory trace achieves an average accuracy of 98.8%±1.16 on the omniglot dataset for five-way one-shot image classification task while requiring 20x fewer trainable parameters in comparison to other state of the art models.

## 1. Introduction

Biological brains are capable of processing massive amounts of information for learning, retaining, and performing cognitive decision making. Moreover, the brains are endowed with the ability to learn continuously and adapt quickly to changes in the inputs or the environment in an energy efficient manner. The extraordinary computational capabilities of biological neural systems has motivated researchers to explore the structural and functional aspects of the brain, in order to build intelligent systems capable of solving complex tasks (Mead, 1990; Rumelhart et al., 1995; Kar, 2016; Hassabis et al., 2017). Using the brain as a source of inspiration, researchers have successfully demonstrated networks with the ability to solve complex learning tasks. For example, convolutional neural networks (Lawrence et al., 1997; Huang et al., 2017) have demonstrated remarkable performance for image recognition tasks, recurrent neural networks (Williams and Zipser, 1989; Gers et al., 2002; Greff et al., 2017; Sak and Senior, 2018) have been able to perform classification, prediction, and anomaly detection on temporal tasks, and few reinforcement learning based systems (Sutton and Barto, 1998; Silver et al., 2016) are able to learn complex cognitive tasks.

While such state-of-the-art networks perform well on narrow sets of well-defined tasks, they are generally not very good at generalizing, and they are not able to learn from few samples (Bengio et al., 2015; Rosenfeld et al., 2018). To address these issues, biological brains have mechanisms that dynamically adjust its own parameters for learning in new environments. These mechanisms play a key role in reacting and responding to stimulus based on context in a quick and efficient way. It has been observed in many species—from insects to humans—, that, in addition to synaptic plasticity, neuromodulation plays a key role in the facilitation of learning (Decker and McGaugh, 1991). Brains also use neuromodulation to modify neural connectivity in response to inputs and internal states. Neuromodulation is the physiological process by which a given neuron uses one or more neurotransmitters to regulate a population of neurons (Katz and Edwards, 1999). This contrasts with classical synaptic transmission, in which one presynaptic neuron directly influences a single postsynaptic partner. Neuromodulators secreted by a small group of neurons diffuse through large areas of the nervous system thus affecting multiple neurons. Reports have shown that neuromodulation affects synaptic plasticity, neural wiring, and attention (Katz, 1999; Doya, 2002).

In this work, we develop computationally efficient dynamic learning systems inspired from neuromodulatory mechanisms in the brain wherein a modulatory unit regulates the learning according to the context and the internal state of the system. Here, the internal state of the system refers to the activations of the neurons in response to the current input. When we refer to dynamic learning, we focus on the capability of associative learning; where the system learns to discriminate its input based on a context, which can either be internal to the system or triggered by an external input, such as a reinforcement or a modulatory signal. In addition to implementing dynamic learning capabilities, our architecture needs an attention component responsible for meta-learning: its main function is to evaluate when, what, and how much to learn based on the context. Thus one approach toward solving this problem is by incorporating the heterosynaptic (neuromodulatory) mechanisms in conventional neural networks.

Some researchers have incorporated the concept of neuromodulated plasticity into network models for solving tasks in dynamic reward-based scenarios. Soltoggio et al. (2008) proposed an architecture where they introduced the concept of modulatory neurons that enhances or dampens the neural plasticity of the target neurons to boost the memory and learning capabilities. The concept of gated plasticity in Soltoggio et al. (2008) enabled dynamic targeted update of synapses in the network, thus leading to more efficient learning. The work in Miconi et al. (2020), demonstrates that adding neuromodulatory plasticity mechanisms trained using gradient descent exhibit superior performance on reinforcement learning and non-trivial supervised learning tasks like few shot learning, pattern memorization, and image reconstruction. They also explain that self-modifying capabilities in the brain play an important role in learning and adaptation. This work shows that incorporating these learning mechanisms along with an architecture inspired from insects enables learning dynamically and from few samples in a computationally efficient fashion.

The key contributions of this work are:

- Incorporating architectural and functional methods inspired from the insect brain to enable neuromodulatory interactions in conventional neural networks.
- Adaptive local learning rules with built-in attention mechanisms that endow the networks with the capability to learn from few-samples.
- A compartmentalized network architecture akin to the mushroom body in the drospohila to process the information in a scalable and resource efficient way.
- A modified modulatory trace learning rule capable of learning and efficiently processing inputs from the internal state of the system.

## 2. Related Work

### 2.1. Neuromodulated Plasticity in Neural Networks

In the brain, the neurons communicate with each other by releasing neurotransmitters when the axon potential of the neuron reaches a synapse. Depending on the type of the neurotransmitter, the receiving neuron can be in either excitatory or inhibitory state. Neurotransmitters can sometimes cause an electrical signal to be transmitted down the cell (excitatory), whereas in other cases, the neurotransmitter can actually block the signal from continuing, thereby preventing the message from being carried on (inhibitory). Some of the neurotransmitters that have spatially distributed and temporally extended effects on the recipient neurons and circuits, are called Neuromodulators (Katz and Edwards, 1999). The best examples of neuromodulators are dopamine, serotonin, noradrenaline (also called as norepinephrine) and acetylcholine. Doya (2002) hypothesized the role of different neuromodulators in the context of reinforcement learning in the brain. His hypothesis was as followed: Dopamine acts as the global control and learning signal for the network for predicting rewards and reinforcement of actions. Serotonin modulates the balance between the short-term and long-term prediction of rewards. Similarly, noradrenaline modulates the attention mechanism in the network in the sense that it controls the balance between wide exploration and focused execution. Acetylcholine handles the memory and controls memory storage and renewal of memory. It modulates the learning wherein, based on acetylcholine release, learning new tasks and rate of forgetting of previously learned tasks is handled. Following that, there have been several other hypotheses on similar lines (Bargmann, 2012; Pedrosa and Clopath, 2017; Shine et al., 2019) regarding the functional role of the neuromodulators in the brain. Taking inspiration from this, several researchers have incorporated neuromodulation in deep learning frameworks. Kondo (2007) proposed a diffusion-reaction based model using neuromodulation. The neuromodulators via those actions, regulate the synaptic connectivity and strength. This mechanism was able to demonstrate online learning capabilities for mobile robotic control. The concept of neuromodulated spike timing dependent plasticity in spiking neural networks is introduced by Frémaux and Gerstner (2016). These gated plasticity based learning rules show how neuromodulatory signals interact with the neural activity to bias learning and behavior, and respond to novelty. Kolouri et al. (2019) proposes an attention based selective plasticity approach that is based on cholinergic modulation in the brain to address catastrophic forgetting. The central idea in most of the previous works portrays the ability of neuromodulators to impact plasticity predominantly through gating of plasticity and up-regulation of neuronal activity. These features or effects of neuromodulators are observed across multiple species not only including mammals and reptiles, but also insects.

There is an active research aiming to understand how smaller brains can be highly capable of learning and cognition (Montgomer et al., 2018). Despite having brains that are a million times smaller, insects are able to exhibit almost half the distinct cognitive behaviors as that of certain mammals like dolphins (Changizi, 2013; Theobald, 2014) (59 for honeybees compared to 123 for dolphins). For example, bees build honeycombs and operate in swarms via symbolic communication, wasps exhibit chemical communication, termite colonies perform climate control, etc. The neural circuitry found in insect brains is able to exhibit complex cognitive behaviors similar to mammals albeit with a lower resolution and reduced information processing (Lihoreau et al., 2012). Moreover, cognitive ability does not necessarily result from greater numbers of neurons but rather it is the new links between different bundles of neurons that lead to tangible changes in behavior (Chittka and Niven, 2009). Yanguas-Gil et al. (2019) shows that architectures inspired from insect brain are capable of exhibiting context-dependent processing and learning. Therefore, models based on small brains can still offer a good baseline of intelligent tasks in a resource and power efficient manner.

### 2.2. Few Shot Learning

Several real-world application domains like healthcare, robotics, etc., operate on irregular and sparse datasets. To address this issue, few shot learning is becoming a prominent area of research. However, learning and adapting from few examples is very challenging. The conventional approaches for image classification involving convolutional neural networks trained using backpropagation are unable to offer a satisfactory solution for learning new concepts rapidly from little data. Hence, there have been few works that were particularly inclined toward solving this problem and have been able to achieve good performance on few shot learning tasks. The Siamese network model (Koch et al., 2015), tries to approach the problem of few shot learning by giving the model two samples and then training it to guess whether the two samples belong to the same category or not. Another approach to the few shot learning task is specified in Matching Networks (Vinyals et al., 2016). Matching Nets use novel attention mechanisms and embedding functions to enable rapid learning and train the network by showing only few examples per class. They train the network by randomly selecting k labeled examples from N classes that haven't previously been trained upon. The task is then to classify a batch of unlabeled examples into one of these N classes. The model proposed in Mishra et al. (2017) currently achieves state of the art performance for few shot learning tasks. The authors introduced temporal convolution (TC) and causal attention layers in the network, wherein the TC layers provides the context over which the attention layers operate.

Apart from the prior specified techniques, researchers have proposed meta-learning based techniques. The work proposed by Santoro et al. (2016) uses a novel sophisticated memory based system. It uses Long Short-Term Memories (LSTMs) as a memory controller that interfaces with the input and outputs through complex memory accesses. The network learns a general strategy for the types of representations it should place into memory and how it should later use these representations for predictions. Recently, Finn et al. (2017) proposed Model-Agnostic Meta Learning for fast adaptation of Deep networks, that introduces a meta learning algorithm that can be trained with any model with gradient descent and can be used to solve a variety of problems like classification,regression and reinforcement learning. Researchers (Doya, 2002; Soltoggio et al., 2008; Miconi et al., 2020) have studied the role of neuromodulatory and heterosynaptic update mechanisms for endowing networks with meta-learning capabilities. In this work, the authors use plasticity based rules to encode the context and drive the update of parameters in the network in conjunction with backpropagation.

## 3. Modulation Inspired Learning Methods

In the proposed work, the efficacy of adding neuromodulation to the neural networks is observed. The first approach couples synaptic local learning rules with error driven modulation to enable learning on the edge. The second approach incorporates neuromodulation in conjunction with backpropagation wherein a context driven modulatory trace regulates the short term plasticity of the connections.

### 3.1. ModNet

The proposed architecture, Modulatory Network (ModNet), Daram et al. (2019) derives its inspiration from the mushroom body in the insects and the learning mechanism is inspired from the neuromodulatory mechanisms in the brain. Neuromodulators closely affect synaptic plasticity, neural wiring and the mechanisms of long term potentiation (LTP) and long-term depression (LTD). The realization that Hebbian learning is not the only way that synapses are modified (Cooper, 2005) has led to growing interest in neuromodulation. Studies on mollusks and insects (Carew et al., 1981; Roberts and Glanzman, 2003) have shown that in addition to Hebbian learning, neuromodulatory mechanisms are also involved with associative learning and synaptic changes.

The learning rule proposed in our architecture derives from the aforementioned heterosynaptic mechanisms and uses the concept of Hebbian plasticity for the synaptic weight update. The pre-synaptic and post-synaptic neurons determine the polarity of change in the connection while the modulatory neurons regulate the rate at which the weight is updated.

**Figure 1:**
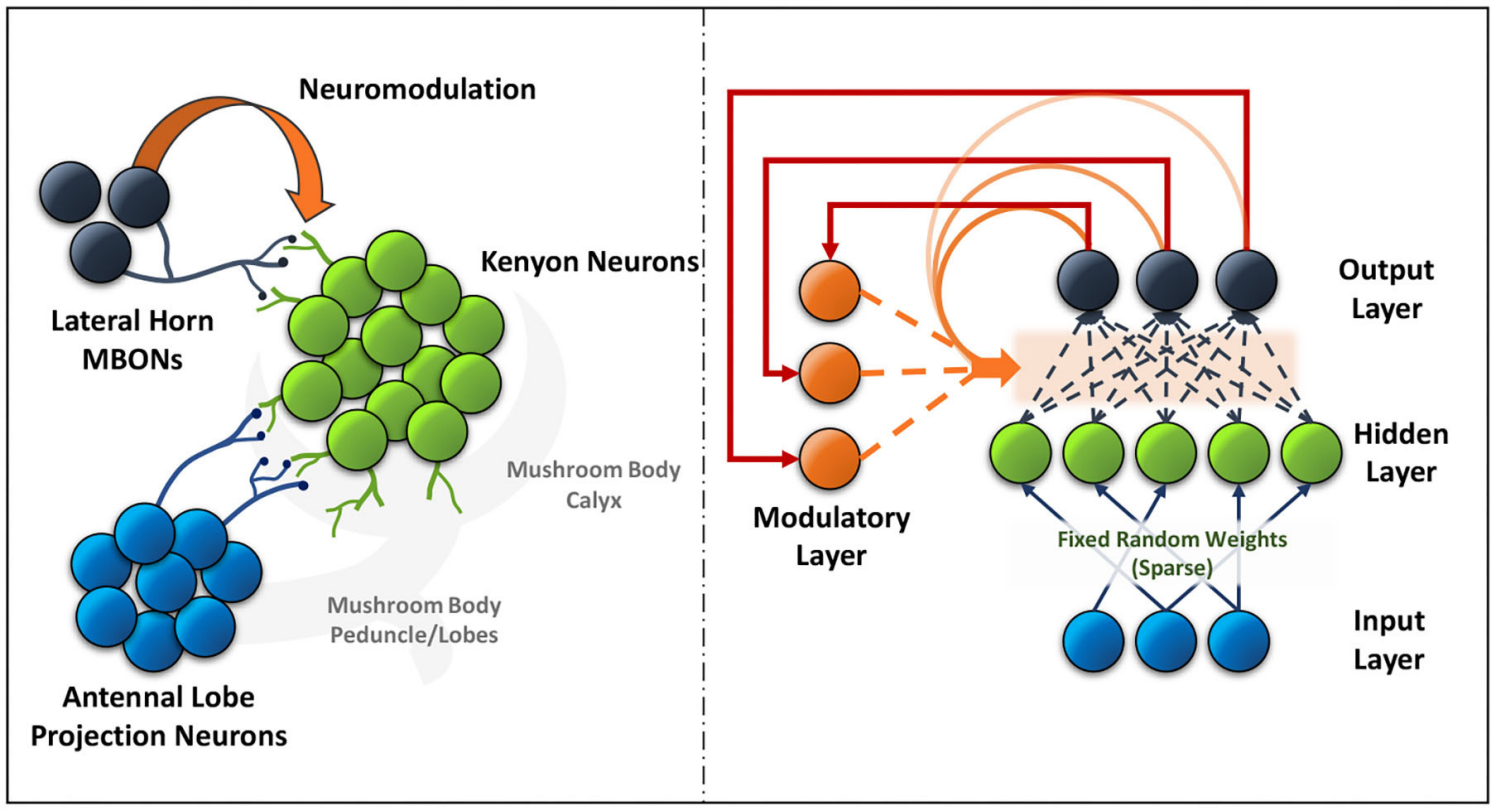
The neural circuit formed between the antennal lobe projection neurons, Kenyon cells and lateral horn MBONs (left) in the mushroom body of insects is akin to the ModNet network architecture proposed (right).

The mushroom body output neurons (MBONs) in the *Drosophila* play a key role in discriminating between stimuli, learning their predictive value and further using that information to modify their behavior (Aso et al., 2014). Additionally, dopaminergic modulation alters the balance within the MBON network for those stimuli. The input layer in the ModNet corresponds to the antennal lobe projection neurons (sensory stimuli). These antennal lobe neurons are sparsely represented in the Kenyon cells and a similar property is used in ModNet, as shown in Figure 1 (Daram et al., 2019), the inputs are randomly projected into a sparse hidden space. Sparsity ensures greater feature separability and distinctive representation of the inputs. The Kenyon cells then converge into multiple MBONs and the plasticity of those connections is regulated by neuromodulation based on stimuli. Similarly, in ModNet, the hidden layer is fully connected to the output layer and the plasticity of those connections is regulated by a modulatory layer. This modulatory layer takes as input the error calculated at the output layer and uses it to regulate the plasticity of the hidden-to-output layer weights.

The network consists of two units, the processing unit which is responsible for learning the features and the distinctive representations, and the neuromodulatory layer which is responsible for learning the context. In the processing unit, the input features are lifted onto a higher dimensional hidden layer which extract the spatial features. These features are processed through sigmoid activation function at the hidden layer and are learned at the output layer. The learning error from the output neurons, with respect to a one-hot encoded label as input, are passed as inputs to the modulatory layer. The modulatory neurons compensate the error by updating the trainable weights from the hidden to output layer neurons. During the training phase, the output neurons have a set of two activations each, namely the standard activation and the modulatory activation. The standard activations are computed as sum of the products of hidden-neuron activations and the hidden-to-output layer weights. The modulatory activations are computed as sums of products of the modulatory inputs to the modulatory weights. The standard and the modulatory activations are calculated as shown in (1) and (2).

(1) $$\begin{array}{l} A_{i}=Sigmoid\left(\sum w_{ij}x_{j}\right) \end{array}$$

(2) $$\begin{array}{l} M_{i}=\sum w_{ij}^{\prime }x_{j}^{\prime } \end{array}$$

where *w*_*ij*_ corresponds to the hidden to output layer weights from the *i*^*th*^ neuron in the output layer to the *j*^*th*^ neuron in the hidden layer, and *x*_*j*_ corresponds to the hidden layer activations. $w_{ij}^{\prime }$ corresponds to modulatory weights from the *i*^*th*^ neuron in the output layer to the *j*^*th*^ neuron in modulatory layer and $x_{j}^{\prime }$ correspond to inputs to modulatory neurons (error computed at the output layer) ,respectively. Once the activations are calculated, the standard and modulatory weights are updated as shown in Equations (3) and (6), respectively.

(3) $$\begin{array}{l} \Delta w_{ij}=Sigmoid\left(\frac{M_{i}}{n_{ij}}\right)\times \delta_{ij} \end{array}$$

The weight update equation has two components with the first part being the magnitude component and δ_*ij*_ being the plasticity and direction term. In (3), *n*_*ij*_ is a scaling parameter that is tuned while training. The plasticity term δ_*ij*_ is realized according to Equation (4),

(4) $$\begin{array}{l} \delta_{ij}=\eta_{ij}\left(\beta_{1}x_{i}x_{j}+\beta_{2}\left(x_{j}-x_{i}\right)+\beta_{3}\right) \end{array}$$

where η_*ij*_ is the adaptive learning parameter that is updated while training, *x*_*i*_ and *x*_*j*_ are the pre and post-synaptic activations, and β_1_, β_2_, and β_3_ are the tunable parameters for the network. The weight update equation has a correlation term β_1_, a difference term β_2_ and a constant term β_3_ as a bias. The constant term allows for update of the synapse even in absence of pre or post synaptic activation. The polarity of η_*ij*_ is changed based on the difference between the activations and the polarity of the connecting weight. Hence in this learning rule, the modulatory component regulates the magnitude of the rate of weight change and the plasticity component determines the sign or direction of the weight update for the given connection. This term selects and strengthens the set of connections contributing toward learning a particular task. The adaptive learning rate is updated according to Equation (5).

(5) $$\begin{array}{l} \eta_{ij}=\eta_{in}\frac{e_{i}}{x_{i}}, \end{array}$$

where η_*in*_ corresponds to the initial value of the learning rate and e_*i*_ and x_*i*_ correspond to the error and the activation at the observed output neuron. In the case when e_*i*_ or x_*i*_ are 0, then the η_*ij*_ is set to η_*in*_. Having the output activation as a divisive factor enables a more optimized rate of change in learning rate based on how far it is from correctly learning the associations. The same equation is also used for updating the modulatory weights, with the error and the activation terms switching positions. This mechanism is similar to attention mechanism in neural networks. The magnitude term that depends on the modulatory interactions, is also affected by the division term which changes the dynamic range of the sigmoid by flattening the curve. The modulatory weights are updated based on Equation (6),

(6) $$\begin{array}{l} \Delta w_{ij}^{\prime }=\eta_{ij}^{\prime }\left(scale\right) \end{array}$$

where $\eta_{ij}^{\prime }$ is an adaptive learning parameter and *scale* is a tunable magnitude parameter. The sign and magnitude of $\eta_{ij}^{\prime }$ is updated as a function of the network response and the output activations. The sign of the learning parameter is directly correlated with the error and the magnitude is increased or decreased based on the value of output activation. The learning rule proposed in ModNet (Equations 3 and 4) consists of Hebbian update coupled with a modulatory regularizer wherein the rate of weight change is either enhanced or dampened with respect to the hidden layer neurons' contribution toward learning. The proposed learning rule enables dynamic learning in the system with exposure to only few samples. But having a sparse hidden layer can lead to many redundant and unused neurons and synapses. Thus, to make the algorithm more efficient, a dynamic attention based mechanism is proposed.

### 3.2. Region Based Attention Mechanism

ModNet essentially shows a baseline network incorporating hetero-synaptic interactions in neural networks. But the network can be designed to be more dynamic to solve complex tasks in a further efficient fashion. Thus a mechanism to add attention in the hidden layer is introduced in this section. The algorithm implements an attention mechanism that distinctly selects populations of excitatory or active neurons while inhibiting and filtering the less active ones. This separation enables efficient distribution of information within the network. Thus, the hidden layer in the ModNet is divided into regions based on a scaling factor α to perform selective filtering and inhibition. This mechanism is adopted while training. The activities of the neurons in different regions are measured and a normalized average of neuronal activities in every region is computed. The activity factors of the regions are computed periodically for every 100 samples. If a_*ik*_ is the activation value of a neuron in k_*th*_ region, then the average activity factor of the region is given by Equation (7).

(7) $$\begin{array}{l} A_{k}=\frac{\frac{1}{a_{max}}\sum a_{ik}}{N} \end{array}$$

where a_*max*_ is the maximum activation value in the given region and N represents the number of hidden neurons in the region. Based on the activity factor, the weights of every region are further updated according to the Equation (8).

(8) $$\begin{array}{l} w_{ik}^{\prime }=w_{ik}-A_{k}\Delta w_{ik}, \end{array}$$

where A_*k*_ controls the rate of change in synaptic strength and boosts the strength of the connections for the more active regions. If the activity (A_*k*_) is less than a threshold value δ, then the weights of the neurons in those regions are further updated according to the Equation (9).

(9) $${w^{\prime }}_{ik}=\{\begin{array}{ll} w_{ik}-|A_{k}\Delta w_{ik}| & {\text{when w}}_{ik}>\text{0}\\ w_{ik}+|A_{k}\Delta w_{ik}| & \text{when }{\text{w}}_{ik}<\text{0}\\ 0 & \text{otherwise} \end{array}$$

As shown in Equation (9), in the regions with lower average activity or smaller activity factor, the connections of the neurons in those regions are inhibited and the weights are made to converge to 0 thereby making those regions sparser. Thus, the region based attention mechanism is able to determine the active and inactive regions during training and is dynamically able to drop out connections and neurons while retaining the performance. This mechanism further enables introducing a more complex and mushroom body inspired architectural formulation known as compartmentalization.

### 3.3. Compartmentalized ModNet

**Figure 2:**
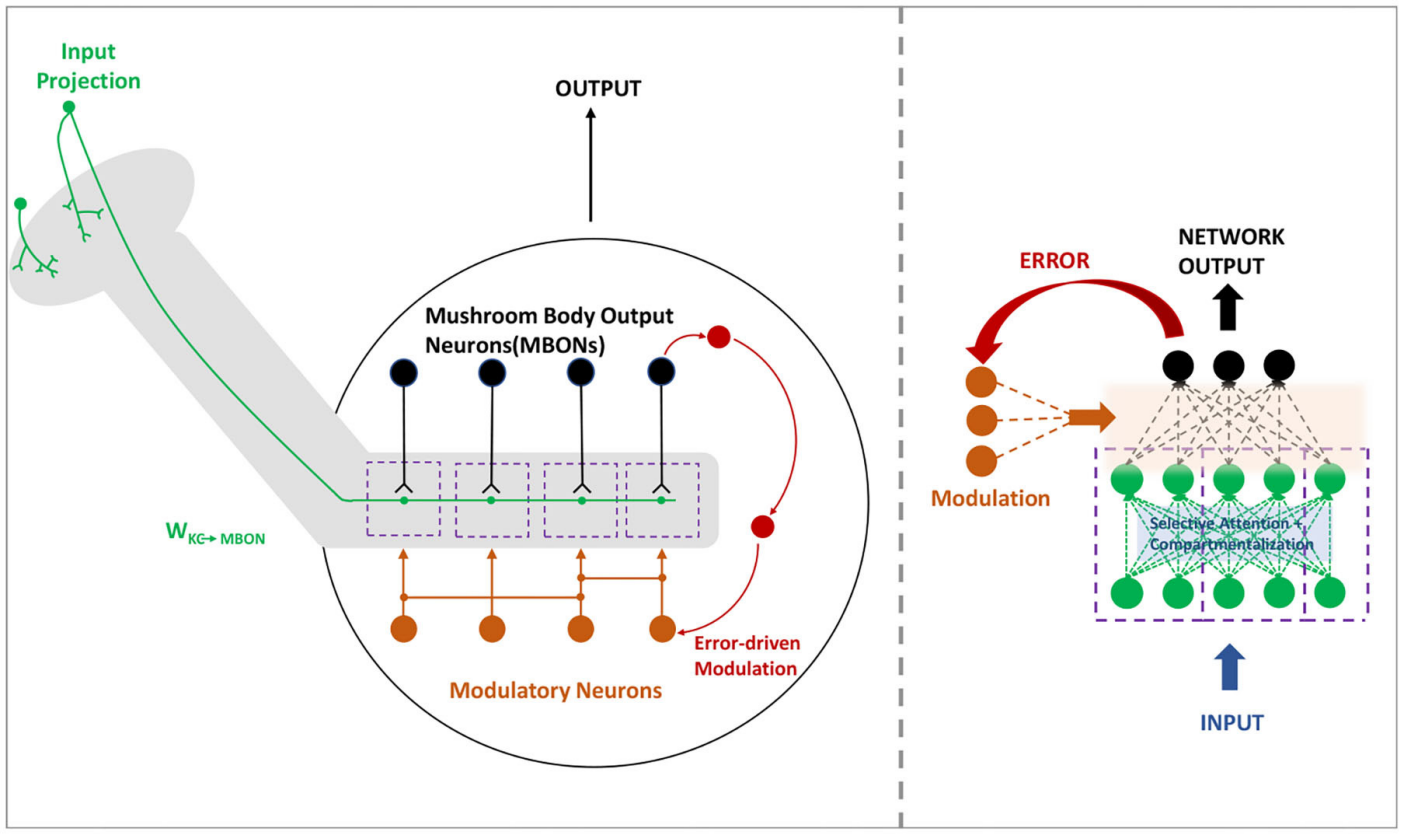
The mushroom body **(left)** in insects are organized in a compartmentalized fashion to encode and bias the behavior. Compartmentalized ModNet architecture **(right)** incorporates this design aspect to perform selective filtering and resource efficient processing of information.

In the mushroom body, the MBONs are able to encode the valence and bias the behavior in a highly compartmentalized fashion (Aso et al., 2014). Every compartment in the mushroom body lobe acts as an independent valuation module with different and changing modular functions. Thus this idea is translated to a deeper version of the region based ModNet, with each region actively learning and acting as independent modules in the network. The core idea behind compartmentalization is to generate a modular network capable of adapting to different tasks with different regions learning to respond to certain tasks with only few data samples. Figure 2 shows the neural architecture of the compartmentalized organization in the mushroom body output neurons in insects.

An additional hidden layer is added to the ModNet architecture. The layers are split into explicit multiple compartments competing to learn the features and eventually gating the less active ones. The net activity factor of each compartment is measured and the connectivity is updated based on the rule presented in Equations (8) and (9). The net activity factor for each compartment is measured as a factor of the activation values of the population of neurons in the respective compartment. The local connections between the neurons in the compartments are trained via local plasticity rules. Unlike gradient descent where the correlations between hierarchical layers are retained by gradients, the problem with having multiple rules across layers causes an issue of uncorrelated knowledge transfer. Hence, an unsupervised covariance based learning rule as shown in Equation (10), is utilized which actively updates the synapses based on correlations between the pre and post synaptic neurons.

(10) $$\begin{array}{l} \Delta w_{ij}=\eta^{\prime }\left[\left(x_{pre}-s1\right)\left(x_{post}-s2\right)\times w\right] \end{array}$$

In Equation (10), s1 and s2 are hyperparameters that define the threshold for activations for the pre and post synaptic neurons to either strengthen or weaken the connection. Moreover, to alleviate the problem of oscillation of neuronal activity due to multiple learning rules, the covariance based learning rule is operated on a slower timescale, thus being updated after every N inputs wherein N can be set as a hyperparameter. The preliminary results demonstrate superior performance than the ModNet architecture on the Fashion-MNIST dataset.

### 3.4. Modulatory Plasticity in Deep Networks

ModNet architecture represents one way of introducing modulatory dynamics to a network and training via modulatory and plastic learning rules. However, the problem with ModNet lies in that being a shallow network, the capabilities of ModNet is limited to solving simpler classification tasks. However, the use of a gradient descent mechanism like backpropagation in neural networks has shown to achieve spectacular results in solving complex tasks. Thus, it would be interesting to add modulatory plasticity to these non-plastic backpropagation based neural networks. This way, not only will the weights be optimized while training via backpropagation but, also the plasticity in each connection is updated via neuromodulation. To test the advantage of having neuromodulated plasticity in the context of lifelong learning, the task of performing efficient one-shot and few-shot learning is considered.

**Figure 3:**
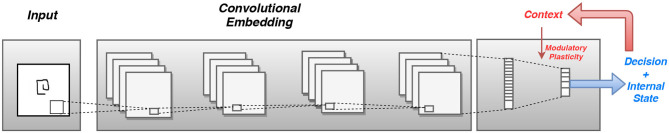
The input is passed through a convolutional embedding and at the final layer, based on the activations from the feature layer and the output layer, the network determines the context and modulates the plasticity of those connections.

Figure 3 shows the concept of incorporating modulatory dynamics into a CNN for solving the task of few shot learning. Thus, to train a modulatory network with backpropagation, a formulation is considered in which each connection has a standard component and a modulatory component. This formulation is inspired from the work in Miconi et al. (2018), wherein the author considers the network to have a fixed and a plastic component for training. However, the plasticity component in Miconi et al. (2018), does not account for the full internal state of the network and thereby does not encode the global context while updating weights in the network. Hence, the connection between any neuron *i* in layer *l* and another neuron *j* in layer *l-1* will have a regular connecting weight w_*ij*_ which constitutes the standard component, and a modulatory trace Mod_*ij*_ which changes based on the current inputs and outputs to the network. Thus the total weight of the connection between any two neurons is given by the sum of the regular weight and the modulatory trace. This is specified in Equation (11).

(11) $$\begin{array}{l} W_{tot}=w_{ij}+\delta_{ij}Mod_{ij}, \end{array}$$

where δ_*ij*_ is the modulatory learning factor that can be either constant for all the connections or different for each. The role of the modulatory trace is to perform heterosynatic weight update of the connections in the network. Hence the output activation is computed as a sum of products passed through a non-linear activation function of the input activations and the W_*tot*_ as represented in Equation (12).

(12) $$\begin{array}{l} x_{out}=\sigma \left(\underset{inputs}{\sum }\left[w_{ij}+\delta_{ij}Mod_{ij}\right]x_{in}\right), \end{array}$$

where x_*out*_ corresponds to the output activation value and x_*in*_ corresponds to the input activations feeding into the output neuron. Here, σ corresponds to the non-linear activation function and the inputs correspond to the input activations from the previous layer.

The modulatory trace is a time dependent quantity for the connections in the network. The trace is updated based on the input and the output activations and a modulatory context which is responsible for handling the short term memory in the network. The modulatory trace is computed according to the Equation (13).

(13) $$\begin{array}{l} Mod_{ij}\left(t\right)=Mod_{ij}\left(t-1\right)+\alpha_{ij}\left[x_{out}\left(x_{in}-x_{out}Mod_{ij}\left(t-1\right)\right)\right], \end{array}$$

where Mod_*ij*_(t) is the currently computed trace value and Mod_*ij*_(t-1) corresponds to the initial trace value or the trace value for the previous iteration. The modulatory trace is initialized to zero at the beginning of each epoch or episode. The parameters *w*_*ij*_ and δ_*ij*_ are trained across all the training epochs and episodes. These parameters are updated and optimized using backpropagation during the training process. α_*ij*_ is defined by the Equation (14).

(14) $$\begin{array}{l} \alpha_{ij}=\gamma \sigma^{\prime }\left(\left[\underset{outputs}{\sum }x_{out}\right]/n\right), \end{array}$$

where σ′ is the non-linear activation function, sigmoid in this case and n is the number of output neurons in the layer and γ is a hyperparameter to regulate the rate of update. The outputs correspond to all the output activations. The α term appears as the modulatory context term which evaluates the network response to encode the global context to the trace.

The parameter α determines the speed at which new information is incorporated into the trace and the plastic component of the network while δ determines the magnitude of effect of the trace on the respective connection. The Mod_*ij*_ term or the trace is accumulated over time but gated by the modulatory context term and the output activation. The Mod_*ij*_ term is an episodic quantity, in the sense that it is reset to zero after every training episode. The modulatory context term and the weights are lifetime quantities as they are updated throughout the training procedure. The modulatory trace in the output layer evaluates the internal state of the system allowing for stable memories, thus enabling the connections to learn the associations between the inputs during the episode. This corresponds to the short term effect of the trace while training during the episode. The modulatory learning factor regulates the long term effect of the trace on the connections in the sense that, being a lifetime parameter, the context term encodes how much, the heterosynaptic update is required for the given task or episode. The modulatory context term reinforces the connectivity and quickly updates the weights in the final layer to adapt to the newer inputs and thus differentiate between the incoming input features from n selected classes in a n-way k-shot learning scenario. This learning mechanism is thus able to perform competitively on the few shot learning task on the Omniglot dataset. This can be attributed to the long term and short term effects of the heterosynaptic mechanisms on the network that are responsible for understanding the context and regulating the response to the encoded context.

## 4. Results and Analysis

In the proposed work, the ModNet architecture and the Modulatory trace learning method are evaluated and tested on different datasets based on the applications. The ModNet architecture is tested on the MNIST (LeCun et al., 1998) and the Fashion-MNIST (Xiao et al., 2017) image recognition benchmarks for classification, and the Modulatory trace learning method is verified on the Omniglot dataset (Lake et al., 2015) for few-shot classification task. In this section, the proposed network architectures are analyzed for performance and efficiency.

### 4.1. Image Classification Tasks

The ModNet architecture coupled with the learning and architectural mechanisms proposed in Sections 3.1-3.3 are evaluated for resource efficient dynamic learning on image classification benchmarks.

#### 4.1.1. Benchmarks and Experiment Setup

Two spatial datasets are used to study the capability of ModNet in learning from few samples. Both the datasets consist of 60,000 training and 10,000 testing images. The MNIST dataset images are normalized and reshaped from 28 ×28 to 10×10. The images from the Fashion-MNIST dataset are only normalized and not reshaped to 10×10 to prevent loss of useful features.

Table 1 shows the network setup for evaluating the ModNet architecture. The ModNet architecture configuration is set to 100 (input) ×1,000 (hidden) × 10 when testing on the MNIST dataset. The input layer size is updated to 784 while testing on the Fashion-MNIST dataset. In the case of Compartmentalized-ModNet, an additional hidden layer of size 1,000 is added to the ModNet network and analyzed.

**Table 1:** Experimental setup and hyperparameters for evaluating the ModNet architecture.

**Network features**	**ModNet**			
Datasets	MNIST		Fashion-MNIST	
Network size	100 × 1,000 × 10		784 × 1,000 × 10	
Hyperparameters	β1	β2	β3	η
	0.1	0.2	0.001	0.001-0.01
Training epochs/Episodes	2			
Runs	10			

#### 4.1.2. ModNet Performance

**Figure 4:**
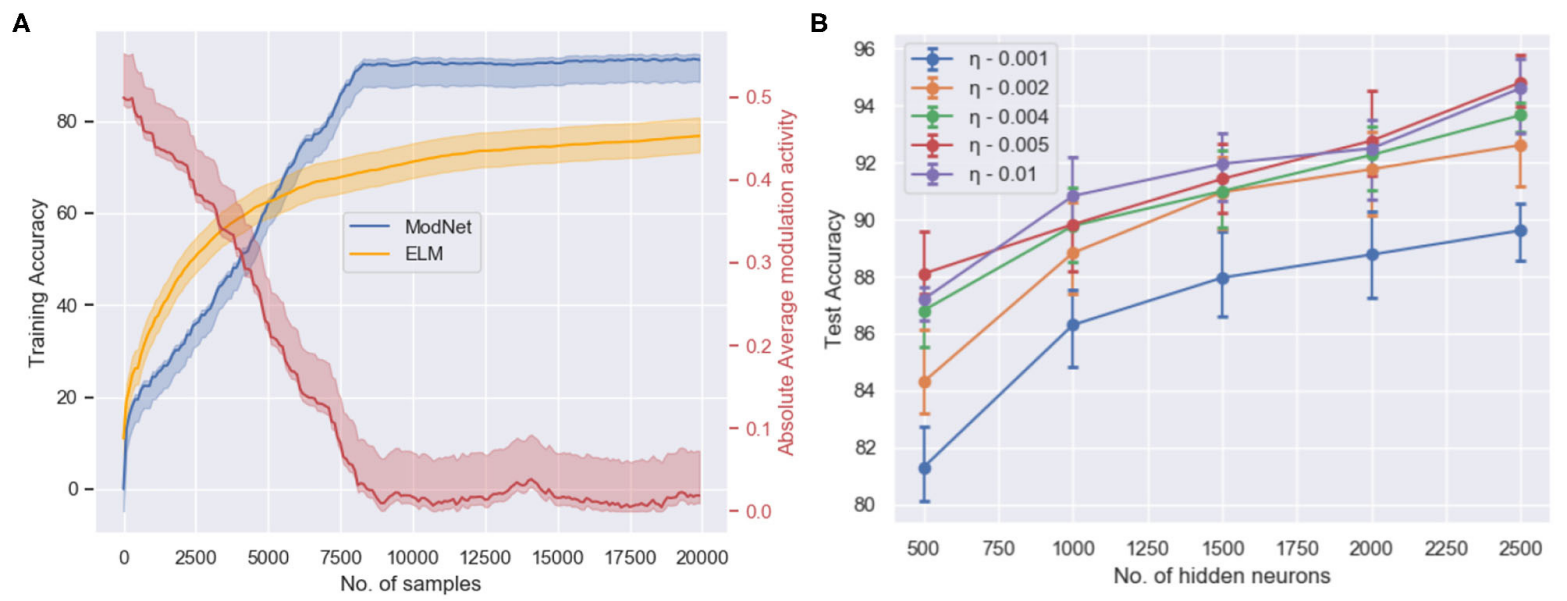
**(A)** The training accuracy (blue) on the MNIST dataset with respect to the number of trained samples and the change in absolute modulatory activation values (red) for a hidden layer of size 1,000. **(B)** Test accuracy with respect to the number of hidden layer neurons and the initialized value of the adaptive learning parameter when trained for 2 epochs on the MNIST dataset for the ModNet architecture.

As observed in Figure 4, the network is able to attain a test accuracy of ~91% while training for just 2 epochs on the MNIST dataset and ~81% on the Fashion-MNIST dataset. Figure 4A shows the training performance of ModNet with respect to the number of samples shown. The network is compared to a similar shallow random projection based network, called the Extreme Learning Machine (ELM) (Huang et al., 2004) which is unable to learn quickly as ModNet. This is a result of the effect of modulatory activations on weights in the network which initially try to reward and penalize neurons at a much higher rate as compared to the later stages when learning begins to saturate. Figure 4B shows how higher dimensionality leads to better performance, which correlates well with the biological counterpart in which the mushroom body projection neurons are lifted in the Kenyon cells. Higher dimensionality in the hidden layer results in a greater feature separation amongst the inputs.

On the contrary, the network performance does not improve significantly after increasing the size of the hidden layer past 2,500 neurons. However, the larger network performs better if it is trained longer. This is a result of the inability of the shallow network to capture certain distinctive features that could be realized by increasing the depth of the model. Therefore, the compartmentalized topology of ModNet and the attention mechanism are evaluated to address the depth of the network and efficient processing for shallower networks, respectively.

#### 4.1.3. Exploiting Sparsity for Efficient Processing

**Figure 5:**
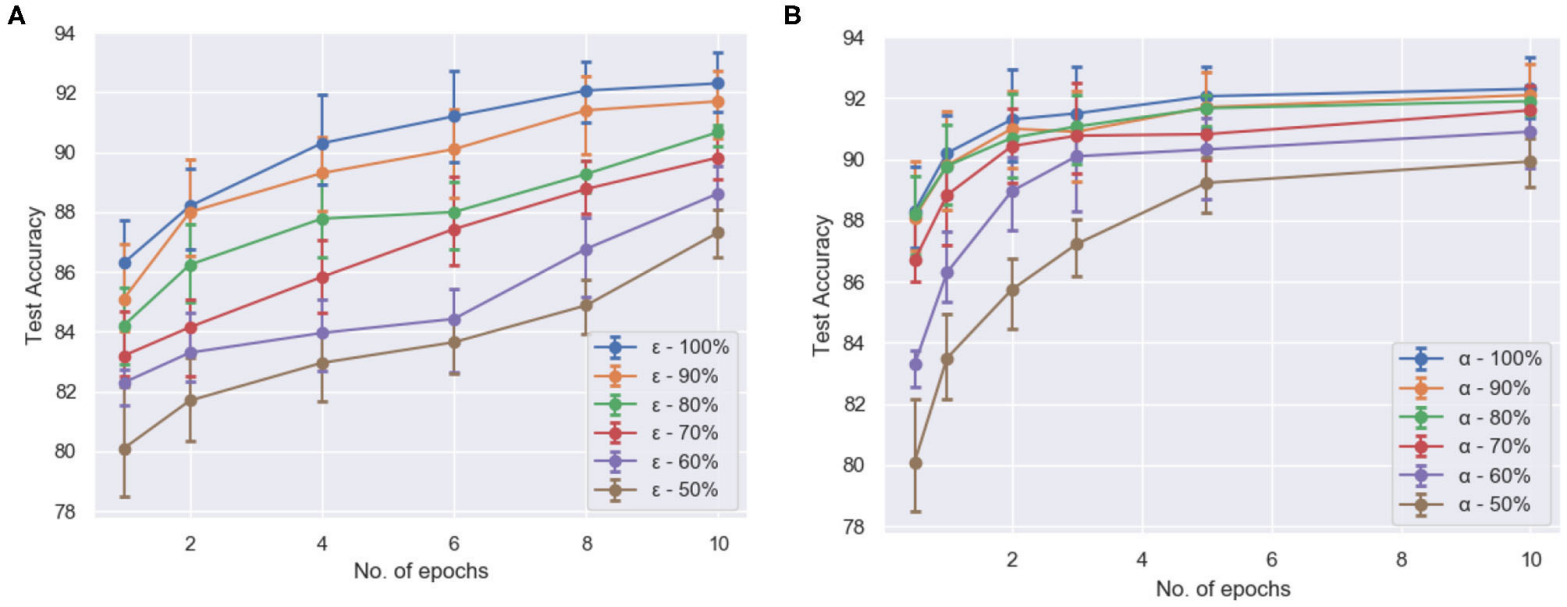
The test accuracy (averaged over 5 runs each) **(A)** of compartmentalized ModNet with respect to the number of epochs and *epsilon* (initial % of randomly selected active neurons) for performing classification on the Fashion-MNIST dataset and **(B)** of ModNet with attention (without compartmentalization) with respect to the number of epochs and the initial percentage of the active regions (α) on the MNIST dataset.

To test the efficacy of compartmentalization with attention on ModNet, the network is re-evaluated on the Fashion-MNIST dataset. For the compartmentalized network, another hidden layer is added with the network configuration being 784 (input) × 1,000 (hidden1) × 1,000 (hidden2) × 10 and selective attention in the compartments in the hidden layers. The network is able to classify with an accuracy of ~91% on the Fashion-MNIST model. Figure 5A shows how selective filtering using attention with compartmentalization and gating allows efficient processing. Turning off least responding compartments and keeping only active compartments while training the network thus ensures greater resource and power efficiency.

**Figure 6:**
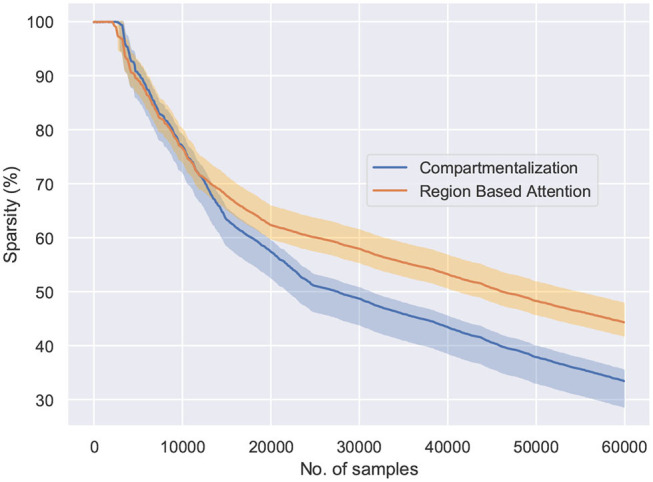
The behavior of the network connectivity with respect to the two plasticity topologies when trained on the MNIST dataset. Compartmentalization induces greater sparsity in the network as a result of increased parameters and finds the optimal sparsity configuration for the given task.

**Figure 7:**
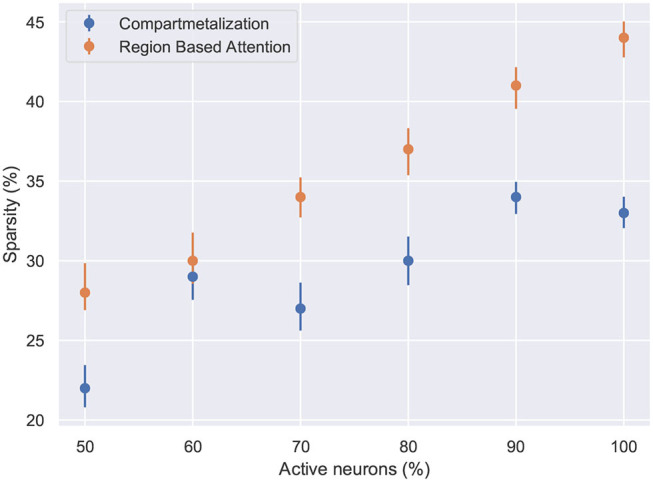
The network sparsity observed after one epoch with respect to the number of active neurons in the network. Compartmentalization is able to find an optimal space despite the variance in the network inactivity and it is observed that the optimal sparsity is achieved at ≈ 30% for compartmentalized ModNet and ≈ 40% for ModNet with attention (Evaluated on the MNIST dataset).

Figure 5B shows the effect of region based attention mechanism and sparse initialization on the performance for the shallow ModNet architecture. We can observe similar performance for a 20–30% sparsely gated network in comparison to the fully connected network. However, other than sparse initialization, the attention mechanism also induces sparsity into the network. To find out the optimal sparsity for the network to perform comparably to the fully connected counterpart, and realize the effect of the aforementioned mechanisms, further analysis is performed as shown in Figures 6, 7.

Figure 6 shows the percentage of connections pruned while training using attention and compartmentalized topologies. Compartmentalization induces greater sparsity as the network has more connections, and a modular topology leads to compartments shutting off after falling below minimum activity threshold. Moreover, to observe the behavior of these mechanisms for already sparse or gated networks, the network sparsity after one epoch for these networks is observed. Based on the results in Figure 7, the optimal sparsity point is observed to be ≈ 30% for compartmentalized ModNet and ≈ 40% for region based attention mechanism. This demonstrates the efficient adaptation aspect of the proposed learning topologies. The learning mechanisms adapt to the different network initializations and try to converge to the optimal sparsity point necessary for performing the task without degrading the performance. However, increased sparsity also affects the network performance in other aspects. Thus, an ablation study is conducted to observe the problems and advantages of each of the proposed mechanisms.

#### 4.1.4. Ablation Study

Table 2 presents the ablation study to demonstrate the efficacy of incorporating different mechanisms in addressing learning from few samples. The results shown in the table are averaged over 10 runs and the number of samples to reach initial convergence are an approximated average of the values over the runs. Coupling attention with the modulatory learning rule ensures reaching convergence quickly while saving on resources by increasing sparsity in the network. As discussed in section 4.1.3, compartmentalization is able to make the network more sparse, but it takes longer to converge as compared to the shallow counterparts. This is also attributed to increased depth and learning using multiple learning rules. However, it compensates by showing improved performance. Enforcing sparsity in the network enables better distribution of information and thereby compartmentalization can be extended to a multi task learning scenario with different subsets of compartments responding to different tasks.

**Table 2:** Ablation study of the proposed learning methods for ModNet on the MNIST and the Fashion-MNIST datasets.

**Network**	**Datasets**					
	**MNIST**			**F-MNIST**		
	**Samples^**a**^**	**Train Acc^**b**^**	**Test Acc^**c**^**	**Samples**	**Train Acc**	**Test Acc**
ModNet	8,000	92.87	87.25	12,000	80.87	74.13
ModNet + Attention	9,500	91.46	86.47	13,000	78.65	72.93
ModNet + Attention + Compartment	11,000	93.82	90.86	15,500	90.85	84.73
ModNet + Sparsity*	14,000	91.39	85.82	23,000	78.76	74.27

* *The network is initialized with 30% sparse input and output connections*.

a *Refers to the number of samples to reach initial convergence*.

b *The mean training accuracy observed where the network initially converges*.

c *The test accuracy of the network when trained for the number of samples specified*.

### 4.2. Few Shot Learning Tasks

One criticism of ModNet and the proposed learning mechanisms is regarding their ability to perform well on larger and more complex problems. Therefore, to evaluate the advantages of using heterosynaptic interactions for complex tasks, we select the few shot learning task.

#### 4.2.1. Benchmarks and Experimental Setup

**Figure 8:**
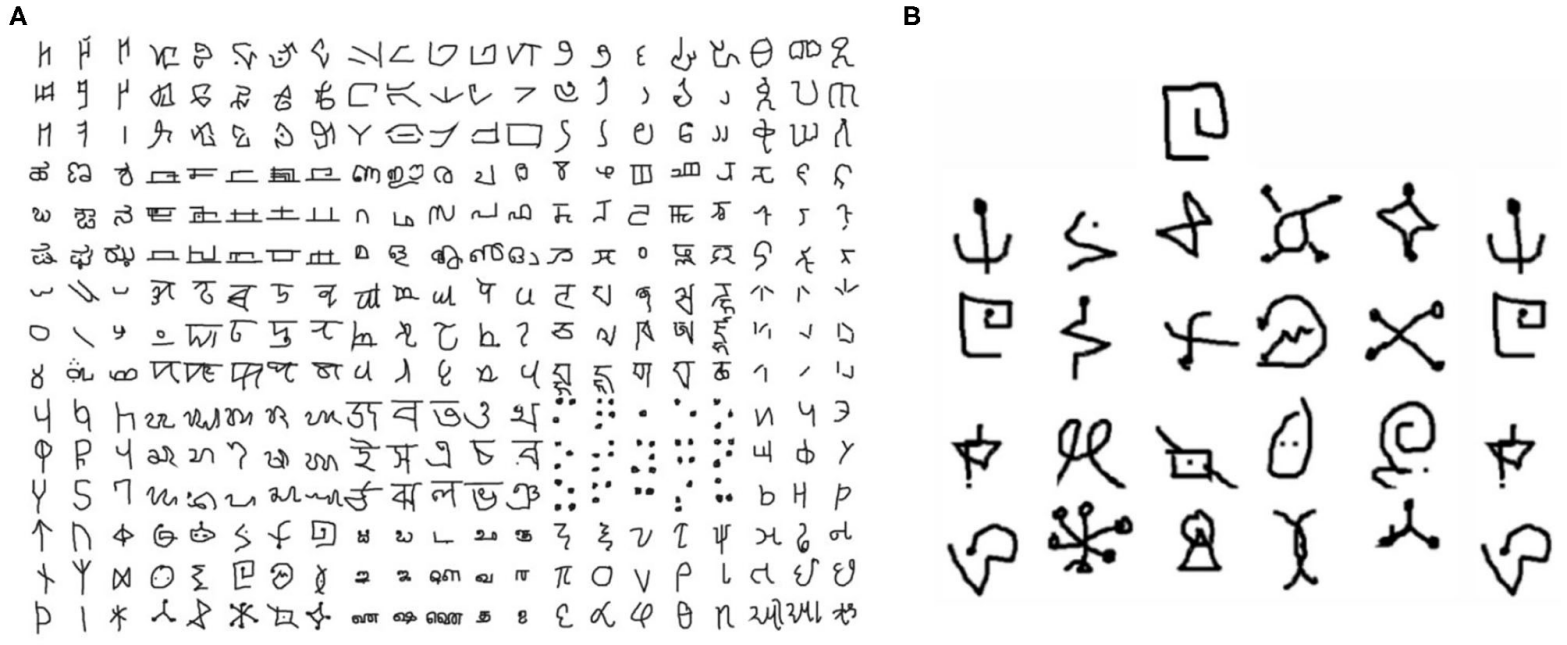
**(A)** A snapshot of some of the classes in the Omniglot dataset. **(B)** The sample N way, K-shot problem set. In this figure N is 20 and K is 1. The network should be capable to classify the unlabeled example from the N character classes.

The modulatory trace learning rule is tested for few shot learning on the Omniglot corpora. The Omniglot dataset Lake et al. (2015), as shown in Figure 8 contains examples from 50 alphabets ranging from well-established international languages to lesser known local dialects. It consists of a total of 1,623 characters with letters from each alphabet varying from about 15 to upwards of 40 characters. Each of these are hand drawn by 20 different people. Moreover, each character in Omniglot is a 105 × 105 binary image. Thus the dataset has 1,623 classes with 20 (105 × 105 images) examples per class.

**Figure 9:**
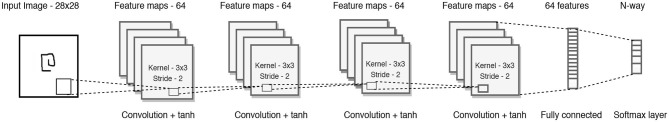
The convolutional architecture used as the baseline. The modulatory trace is applied to the weights connecting the fully connected feature vector to the N-way softmax layer.

Figure 9 shows the baseline CNN architecture used for few shot learning task. It consists of a stack of modules, each of which is a 3 × 3 convolution with 64 filters followed by batch normalization, a ReLU non-linear activation function, and a 2 × 2 max-pooling layer. The images are resized to 28 × 28 so that, when 4 modules are stacked, the resulting feature map is 1 × 1 × 64. This output is a 64 sized vector which then feeds into a N-way softmax layer. Concurrently, the label of the target character is fed as a one-hot encoded value to the softmax layer, guiding the correct output when a label is present.

The task is modeled as an N-way, k-shot classification setup, similar to most of the previous works for few shot classification. This problem thus can be formalized as follows: pick N unseen character classes and K examples of each class from those N, independent of the alphabet and let that set be N, K (Vinyals et al., 2016) as shown in Figure 8B. Each of these instances together with the class labels (from 1 to N) are presented to the model. Then a new unlabeled instance from one of the N classes is presented to the model. The model's performance is defined as the model's accuracy in classifying this unlabeled example. The baseline configuration is tested for N = 5 and K = 1(five way, one shot learning).

The effect of modulatory mechanisms in deeper networks is evaluated for the task of few shot learning on the omniglot dataset (Lake et al., 2015). Modulatory plasticity is introduced in the baseline architecture in Figure 9 for the weights connecting the final feature layer to the softmax layer. The rest of the convolutional embedding does not have modulatory plasticity associated to it. Thus, across the training episodes, the convolutional architecture is expected to learn an adequate discriminant between arbitrary handwritten characters. Meanwhile, the weights between the convolutional network and the softmax should learn to memorize associations between observed patterns and outputs, which are directly influenced by the modulatory context and the labels. Table 3 lists details on the network configuration and hyperparameters used for few-shot learning task. Similar to previous works (Finn et al., 2017; Mishra et al., 2017; Miconi et al., 2018), the dataset is divided into 1,523 classes for training and 100 classes for testing. The network is trained using an Adam optimizer with a learning rate of 3 × 10^−5^, multiplied by 2/3 every 100,000 episodes) over 500,000 episodes. To evaluate the final performance, multiple models with different random seed initializations are trained and then tested on the previously unseen classes for 200 episodes.

**Table 3:** Experimental setup and hyperparameters for evaluating the modulatory trace learning rule.

**Network features**	**Modulatory trace learning**		
Datasets	Omniglot		
Network size	4 Conv, 1 FC		
Hyperparameters	LR	γ	δ
	3 × 10^-5^	0.02	0.01
Training episodes	500,000		
Runs	5		
Data augmentation	Rotations (Multiples of 90^o^)		

#### 4.2.2. Performance Analysis

**Figure 10:**
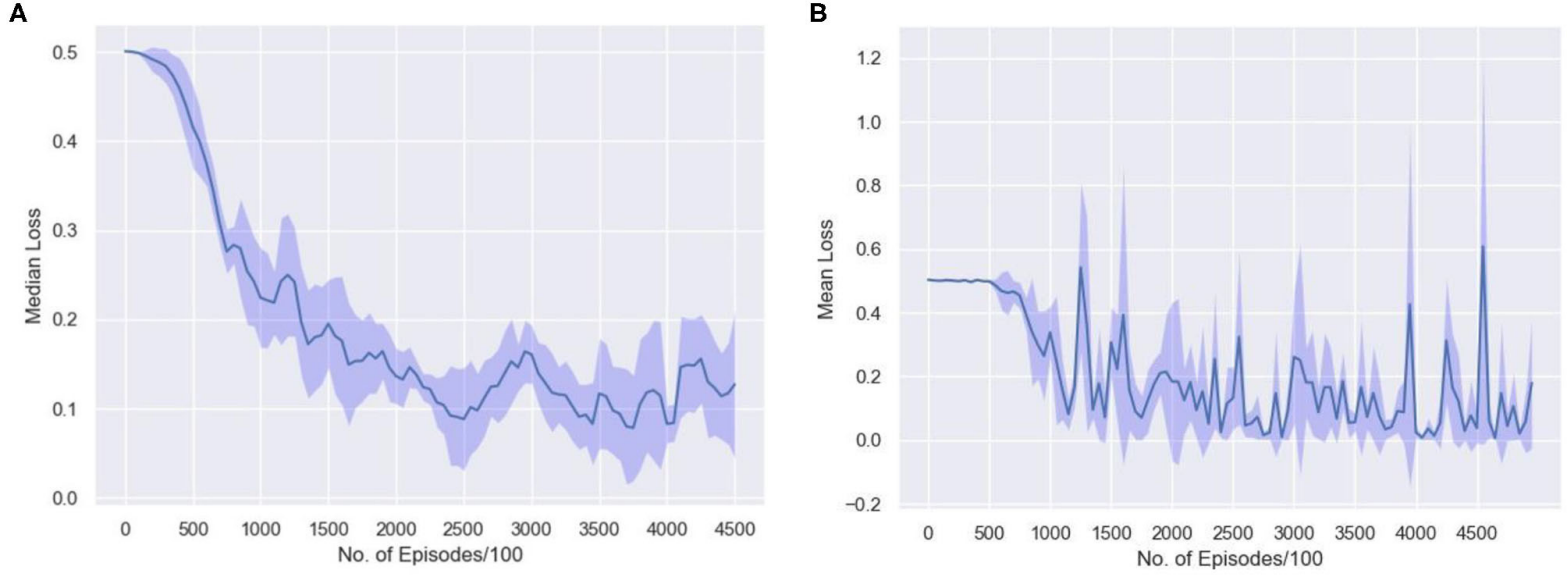
The average loss is computed in the interval of 100 episodes along milestones set after every 50,000 episodes. The **(A)** moving average of the median loss and **(B)** the mean loss along the milestone axis, across 10 runs with different random initializations are shown.

To understand how few shot learning networks train and how the loss varies when presented with new distributions of inputs in a N-way,k-shot learning task, the moving average of median loss, and the mean loss across the training procedure is visualized. Figure 10A shows the moving average of the median loss across multiple runs. The mean is calculated across points after different milestones spaced evenly for every 50,000 episodes. The average loss is computed and saved after 100 episodes to create a net loss matrix. The median of losses along the milestone axis is computed and the moving average along those median values is plotted. The overall median loss decreases as the network is trained longer. This plot shows how few shot learning task does not have a standard loss gradient when presented with more samples as the training involves accessing new set of inputs every time.

Figure 10B represents how the loss actually varies during the training phase. The repeated spikes in the loss is due to the way the network is trained. Since, there are 1,523 classes and 5 classes are randomly selected and moreover, randomly permuted and rotated, the high loss scenarios occur mostly when the randomly selected samples are almost similar. Thus, the loss is higher in some episodes as a result of being trained on new and difficult episodes. The 5 samples have very few distinctive features and thus learning the associations might be difficult in that case. This type of learning is consistent across multiple runs with different random initializations as shown in the plot.

**Figure 11:**
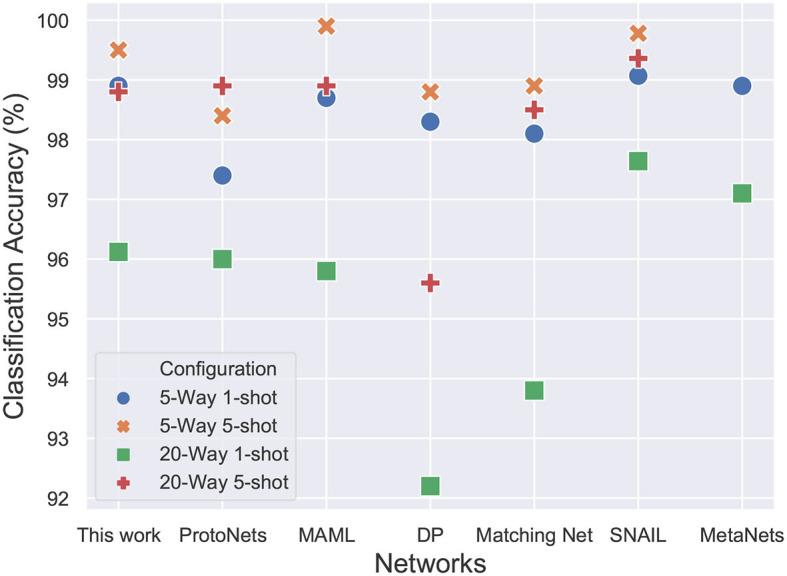
20-way and 5-way, 1-shot and 5-shot classification accuracies on Omniglot dataset. The proposed model achieves comparable accuracy to state-of-the-art SNAIL architecture while requiring 20x fewer trainable parameters and fewer training episodes as compared to MAML.

The results in Figure 11 show the performance of the proposed network in comparison to the accuracies reported in the recent literature. Other than the memory networks, all the other works make use of the baseline convolutional network that has been described previously. The accuracy of the model is almost similar to the computationally intensive MAML (Finn et al., 2017) approach which optimizes for the loss function using gradient descent. The results are almost similar to the Matching networks (Vinyals et al., 2016), Differentiable Plastic networks (Miconi et al., 2018) and Meta networks (Munkhdalai and Yu, 2017). The results reported in SNAIL (Mishra et al., 2017) outperform all the other networks and are currently state of the art but the difference is barely significant. The SNAIL approach trains a whole temporal convolutional layer and causal layers on top of the baseline convolutional embedding leading to a significant increase in the number of parameters. The proposed network performance is near state of the art accuracy for an additional 330 (66× N, with N=5) parameters. The network has a total of 112,065 parameters. The proposed model requires ≈10x fewer training episodes than Differentiable Plastic networks.

#### 4.2.3. Resource Efficiency Through Quantization

To further optimize the resource usage in ModNet, we study the impact of reducing the bit precision. The convolutional embedding is pretrained offline and few-shot learning is performed on a quantized version of the model.

(15) $$PLA(x)=\{\begin{array}{ll} 1, & when\;abs(x)\geq 3\\ 0.06abs(x)+0.815, & when\;1.5\leq x<3\\ 0.443abs(x)+0.24, & when\;0.5\leq abs(x)<1.5\\ 0.924abs(x), & otherwise \end{array}$$

(16) $$Tanh(x)=\{\begin{array}{ll} PLA(x), & when\;x\geq 0\\ -PLA(x), & otherwise \end{array}$$

**Figure 12:**
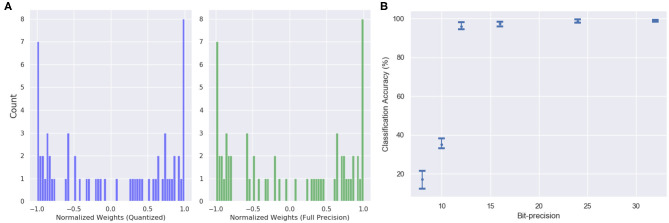
**(A)** The weight distribution of the output feature vector for 16-bit fixed point quantized weights (blue) and 32-bit floating point precision weights (green). **(B)** Variation in network performance with respect to changing bit precision. The performance degradation is minimal for 12-bit precision weights and inputs.

The features in the quantized model include inputs and weights represented in 16-bit fixed-point format with 6 bits for signed integer and 10 bits for fractional part, along with 32-bit partial sum accumulators which are again rounded to 16-bit. The tanh activation is replaced by a piecewise linear approximation as shown in Equations (15) and (16). One interesting observation is that the bit-precision of the output layer made a significant impact on the accuracy. The network performance degraded substantially (from ≈ 98 to 47%) when quantized to 16-bit fixed point. However, the accuracy remained the same as the 32-bit floating point when the output layer is not quantized. Figure 12 shows the weight distribution of the output feature vector for the quantized and the 32-bit operations. The plot shows that the quantized version is able to generate almost similar output feature activations with low quantization error of 1.254%.

## 5. Conclusions

This work shows that incorporating neuromodulatory mechanisms in neural networks is effective toward realizing dynamic learning systems that are able to learn associations and discrimination in the input stream, based on a context. The main contributions of this work are the design of an architecture and adaptive learning rules to introduce modulatory dynamics in the neural networks. The proposed architecture uses simple plasticity rules with a modulatory control mechanism for learning instead of using backpropagation. The ModNet architecture is capable of learning quickly and from fewer samples. This work also introduces a neuromodulation-inspired training technique to self-modify weights in a network. These simple plasticity mechanisms when combined with conventional gradient descent approaches are able to solve non-trivial tasks like few shot learning of different human written characters.

ModNet and compartmentalized ModNet, despite being shallow networks are able to train on the complete MNIST and Fashion-MNIST dataset in just 2 epochs and reach convergence within 8,000 samples of MNIST with 91% accuracy and 12,000 samples of Fashion-MNIST with 89% accuracy. Furthermore, the modulatory trace learning rule in tandem with backpropagation shows accuracy of 98.8% with a 95% confidence interval on the non-trivial few shot learning task on the Omniglot dataset for an additional 325 trainable parameters. These experiments prove that compact and simple meta learning approaches via neuromodulation can perform as well as current computationally intensive methods. Compartmentalization integrated with multiple local plasticity rules might alleviate catastrophic forgetting in neural networks and enable multi-task learning.

## Data Availability Statement

Publicly available datasets were analyzed in this study. This data can be found here: http://yann.lecun.com/exdb/mnist/, https://github.com/zalandoresearch/fashion-mnist, https://github.com/brendenlake/omniglot.

## Author Contributions

AD developed the theory and performed the experiments and computations. DK and AY-G verified the experiments and supervised the findings in the proposed work. All authors contributed to the final manuscript.

## Conflict of Interest

The authors declare that the research was conducted in the absence of any commercial or financial relationships that could be construed as a potential conflict of interest.
